# How Active Older Adults Manage Their Mobility Needs: Transportation Insights From a Panel of Adults Ages 85+

**DOI:** 10.1093/geroni/igaa057.2071

**Published:** 2020-12-16

**Authors:** Lisa D’Ambrosio, John Rudnik, Chaiwoo Lee, Taylor Patskanick, Julie Miller

**Affiliations:** 1 MIT, Cambridge, Massachusetts, United States; 2 MIT AgeLab, Cambridge, Massachusetts, United States

## Abstract

Research suggests that adults experience tremendous transportation challenges at ages 85 and over. As mobility has been tied to overall health and wellbeing, the implications are dire. In this study, the Lifestyle Leaders were surveyed to understand their experiences with different modes of transportation. Responses to a questionnaire (N = 18) and focus groups (N = 18) indicate that many of the Lifestyle Leaders are still driving and report satisfaction with their ability to get around. However, over the past 10 years, many participants have changed their attitudes toward driving and transportation in general. Some panelists cite issues of physical discomfort, increased time and energy spent planning trips, and an increased sense of dependence. Additional data suggest that decision making processes for selecting transportation modes have changed over time. This presentation will discuss policy and practice implications for older adults, caregivers, and transportation professionals.

